# Attachment and Survival of *Escherichia coli* O157:H7 on In-Shell Hazelnuts

**DOI:** 10.3390/ijerph15061122

**Published:** 2018-05-30

**Authors:** Lingyu Feng, Muhammed S. Muyyarikkandy, Stephanie R. B. Brown, Mary Anne Amalaradjou

**Affiliations:** Department of Animal Science, University of Connecticut, Storrs, CT 06269, USA; Lingyu.feng@gmail.com (L.F.); muhammed.muyyarikkandy@uconn.edu (M.S.M); stephanie.barnes@uconn.edu (S.R.B.B.)

**Keywords:** *Escherichia coli* O157:H7, in-shell hazel nuts, attachment, survival, gene expression, wet inoculation, dry inoculation

## Abstract

The multistate *Escherichia coli* (*E. coli*) O157:H7 outbreak associated with in-shell hazelnuts highlights the pathogen’s ability to involve non-traditional vehicles in foodborne infections. Furthermore, it underscores significant gaps in our knowledge of pathogen survivability and persistence on nuts. Therefore, this study investigated the ability of *E. coli* O157:H7 to attach and survive on in-shell hazelnuts. In-shell hazelnuts were inoculated with a four-strain mixture of *E. coli* O157:H7 at 7.6 log colony forming units (CFU)/nut by wet or dry inoculation, stored at ambient conditions (24 ± 1 °C; 40% ± 3% relative humidity (RH) and sampled for twelve months. For the attachment assay, in-shell hazelnuts were inoculated and the adherent population was enumerated at 30 s^−1^ h following inoculation. Irrespective of the inoculation method, ~5 log CFU of adherent *E. coli* O157:H7 was recovered from the hazelnuts as early as 30 s after inoculation. Conversely, pathogen survival was significantly reduced under dry inoculation with samples being enrichment negative after five months of storage (*p* < 0.05). On the other hand, wet inoculation led to a significantly longer persistence of the pathogen with ~3 log CFU being recovered from the in-shell nuts at 12 months of storage (*p* < 0.05). These results indicate that *E. coli* O157:H7 can survive in significant numbers on in-shell hazelnuts when stored under ambient conditions.

## 1. Introduction

Historically, tree nuts have not been associated with a high risk for bacterial foodborne disease. This is mainly due to the hostile environment they provide to the growth and survival of pathogens [1,2]. Therefore, nut-associated outbreaks have been sporadic in the past. However, recent increases in foodborne illness associated with tree nut consumption have led to an increasing concern regarding the safety of minimally processed foods such as nuts. Among the different foodborne pathogens, *Salmonella* has been frequently associated with nuts and nut products including almonds, peanuts, cashew, Brazil nuts, and walnuts [3,4,5,6,7]. Although traditionally associated with ground beef and leafy green vegetables, the recent outbreak of *Escherichia coli* (*E. coli*) O157:H7 in hazelnuts highlights the propensity of the pathogen to colonize diverse surfaces and involve new vehicles that can transmit foodborne infections [8]. According to the Centers for Disease Control and Prevention (CDC) surveillance of foodborne outbreaks in the U.S. indicated that between the years 1998–2008, 7% of *E. coli* O157:H7 outbreaks were due to consumption of contaminated nuts [9]. In addition to resulting in foodborne illness, the presence of pathogens also leads to food recalls. According to Dey and others [10], between the years of 2003–2011, microbiological contamination accounted for 42% of food recalls, with nuts and edible seeds being the most commonly recalled food product. Taken together, these outbreaks and recalls highlight the increasing role of tree nuts as a vehicle for foodborne pathogens.

The reservoir for *E. coli* O157:H7 is domestic livestock [11]. From this reservoir, the pathogen can get transferred to wild animals, contaminate water used for irrigation, and be transmitted by wind [12,13]. In the case of hazelnuts, harvesting practices create a possible route for contamination from feces of domestic livestock or wild deer [8]. Commercially grown hazelnuts are harvested mechanically by sweeping and depositing the nuts in a narrow windrow in the center of the tree row. This is followed by the use of a harvesting machine that lifts and separates the nuts from the fallen leaves and twigs [14]. This process of harvesting nuts from the orchard floor could serve as a potential route for the transfer of pathogens from the soil to the nuts. Further, dusts generated during tree nut harvesting and processing could also help transmit pathogens on to the nuts [15,16,17]. Following harvest, the in-husk hazelnuts are dried under ambient conditions using a variety of methods, including drying tables, plastic mesh bags, or slotted bins. Once dried, husking is performed to separate the husks from the nuts. These in-shell hazelnuts are then cracked to obtain the kernel or are sold as such [18]. Although low temperatures are recognized as an effective means to prolong hazelnut storage, in-shell nuts can be stored at ambient temperatures (24–26 °C, Relative Humidity (RH) 35–45%; [19,20,21]).

Following inadvertent contamination, pathogens must be able to attach, survive, and persist on the nuts in order to result in a foodborne infection. Successful adhesion and attachment of the pathogen on the epiphytic surface is critical to its survival and persistence [22,23]. The initial adhesion of the bacteria on coming into contact with the plant surface is characterized by weak and reversible binding to the substrate [24]. Following this, a strong irreversible binding (attachment) occurs. Once attached, removal of the pathogen cannot be readily achieved [23,25]. Studies on the attachment of *E. coli* O157:H7 indicate that this enteric pathogen can rapidly adhere and attach to different produce, including peaches, plums, alfalfa sprouts, spinach leaves, lettuce leaves, and cut green pepper [26,27,28,29,30]. Overall, the minimum time required for pathogen attachment ranged from 30 s to 1 h varying with the produce type. Similarly, Cui et al. [31] observed that *E. coli* O157 were adept at attaching to different seeds, including alfalfa, fenugreek, lettuce, and tomato. Additional investigations revealed that this attachment is mediated by the production of pili, fimbriae, and non-fimbrial adhesins [32]. Once firmly attached, pathogens could potentially survive and persist on the epiphytic surface [23].

Given the multiple foodborne outbreaks associated with the consumption of nuts, several studies have been performed to elucidate the survival of *Salmonella*, *E. coli* O157, and *Listeria monocytogenes* on tree nuts [33,34,35,36] These investigations demonstrated that pathogens do not multiply on nuts, but can survive on them for more than a year [35,37,38]. A study performed by Blessington and others [39] investigated the survival of *E. coli* O157:H7 on in-shell walnuts inoculated with 400 CFU/nut. They observed that the pathogen could be recovered from walnut samples even after three months of storage at ambient conditions. Similarly, Brar et al. [38] and Kimber et al. [35] demonstrated that *E. coli* O157 could survive on raw peanuts, pecans, almonds, and pistachios when stored at ambient conditions (22–24 °C, RH 39–64%) for 12 months. Additionally, they observed that the pathogen survival was higher at lower temperatures. Although survival studies have determined the potential for pathogens to survive and persist on the surface of nuts, no data are available for *E. coli* O157 on hazelnuts. Hence, the objective of this study was to evaluate the potential of *E. coli* O157:H7 to attach and survive on in-shell hazelnuts when stored under ambient conditions using a dry and wet inoculation methods. Additionally, expression of genes (*csgA*, *fliA*, *escN*, and *rpoS*) previously identified to play a role in *E. coli* O157 attachment to epiphytic surfaces was also investigated.

## 2. Materials and Methods

### 2.1. Bacterial Isolates and Growth Conditions

*E. coli* O157:H7 strains used in the study were as follows: *E. coli* O157:H7 (Odwalla strain), a clinical isolated from an outbreak associated with apple juice, *E. coli* O157:H7 T-50 (apple isolate); *E. coli* O157:H7 7927, a clinical isolate from an outbreak associated with apple cider, and *E. coli* O157:H7 EDL933, a clinical isolate from an outbreak associated with ground beef. In order to selectively enumerate pathogen populations on the nuts, a stepwise procedure was used to isolate mutants of all strains that were able to grow in media supplemented with nalidixic acid (NA; Sigma-Aldrich, St. Louis, MO, USA; 50 μg/mL; [35]). All bacteriological media used in the study were procured from Difco (Becton, Dickson and Company, Franklin Lakes, NJ, USA).

### 2.2. Preparation of Inoculum

Each strain was cultured separately in 10 mL of sterile Tryptic soy broth (TSB, BD Difco, Becton, Dickson and Company, Sparks, MD, USA) containing NA (50 µg/mL) at 37 °C for 24 h with agitation (100 rpm). Cultures were then transferred for 24 h period onto Tryptic soy agar (TSA; Difco) plates containing NA (50 µg/mL; TSAN) to produce a bacterial lawn. To prepare the inoculum, sterile buffered peptone water (BPW, Difco) was added to each plate and bacterial cells were loosened with a sterile spreader. The approximate bacterial count in each culture was determined spectrophotometrically. Equal volumes containing approximately equal populations from each of the five strains were combined to make the pathogen cocktail. The bacterial count in each culture and the cocktail was determined by dilution and plating on TSAN [35,38].

### 2.3. Nuts

Raw in-shell hazelnuts were obtained from a commercial sheller in Oregon. The nuts were sorted to remove any hazelnuts that were split or cracked. Prior to their use in the following experiments, nuts were sampled, tested and confirmed for the absence of *E. coli* O157:H7 following enrichment and selective isolation as described in the Food and Drug Administration (FDA) Bacteriological Analytical Manual. All nuts were stored at ambient conditions (24 ± 1 °C; 40% ± 3% RH) for less than one month prior to use.

### 2.4. Wet Inoculation of In-Shell Hazelnuts and Storage Conditions

In-shell hazelnuts were inoculated as previously described for in-shell walnuts by Blessington et al. [39] and Uesugi et al. [37]. Briefly, in-shell hazelnuts (400 g) were weighed into a sterile bag, and inoculated with the cocktail (25 mL). The bag was then sealed, shaken, and rubbed by hand for 2 min. Then, the inoculated hazelnuts were spread on to four layers of filter paper to drain the excess liquid and dried under ambient conditions for 24 h. Inoculum levels were determined on hazelnuts immediately after inoculation and after drying, as described below. Following the initial drying, in-shell hazelnuts were placed in sterile plastic bags and manually mixed by shaking for 2 min. For the survival study, inoculated nuts were stored in unsealed bags within closed containers held at ambient conditions (24 ± 1 °C; 40% ± 3% RH) for 12 months [39]. To enumerate pathogen survival, nuts were sampled on day 0, 1, 3, 5, 7, 21, 30, 60, 90, 120, 150, 180, 210, 240, 270, 300, and 360 of storage.

### 2.5. Preparation of Dry Inoculum

The pathogen cocktail was prepared as described in Section 3.2. The prepared inoculum was mixed with sand (fine white silicon dioxide, Fisher Scientific, Waltham, MA, USA) at a ratio of 17.5 mL per 100 g of sand in a zippered polyethylene bag. The bag was then sealed and the inoculated sand was massaged by hand for 2 min. The inoculated mixture was then transferred onto filter papers placed on a sterile baking sheet and dried for 24 h in an incubator set at 40 °C. After drying, inoculated sand was transferred to a zippered bag and mixed by hand from the outside to break up any clumps [15]. The dried inoculated sand was used to inoculate the hazelnuts under Section 2.6. Inoculum levels in the sand were determined immediately after inoculation and after drying for 24 h.

### 2.6. Dry Inoculation of In-Shell Hazelnuts and Storage Conditions

In-shell hazelnuts were inoculated by mixing 25 g of inoculated sand and 200 g of nuts in a zippered polyethylene bag [15]. The sealed bag was manually mixed by rubbing and shaking for 2 min. Following the inoculation, sand was separated from the nuts by shaking in a sterile sieve (U.S. standard #12 testing sieve, 1.7-mm openings, Fisher Scientific, Hampton, NH, USA) for 1 min. Inoculated nuts were then pooled in a zippered bag, manually mixed, and stored in unsealed bags within closed containers held at ambient conditions (24 ± 1 °C; 40% ± 3% RH) for 12 months. Nuts were sampled at designated times throughout the storage, as described in Section 2.4.

### 2.7. E. coli O157:H7 Attachment Assays

For assessment of *E. coli* O157:H7 attachment on in-shell hazelnuts, a modification of the protocol employed to study pathogen attachment on alfalfa sprouts and stone fruits was employed [26,28]. Inoculum was prepared as previously described. For the wet inoculation procedure, hazelnuts were individually dipped in sterile bags containing 10 mL of inoculum for a duration 0, 30 s, 1 min, 2 min, and 1 h. At the end of each dipping time, each hazelnut was washed in 20 mL of BPW by gently shaking the bag for 30 s. Each nut was washed three times and placed in 10 mL of BPW for further microbiological analysis. In the case of the dry inoculation method, individual hazelnuts were dipped in sterile bags containing 10 g of inoculated sand for a duration of 0–1 h and processed as described above to enumerate attached *E. coli* O157:H7 populations. For the wet and dry inoculation experiments, six nuts were sampled at each sampling time and the entire experiment was repeated two times.

### 2.8. Microbiological Analysis

To evaluate pathogen attachment and survival, inoculated in-shell hazelnuts were individually transferred to 10 mL of BPW in a sterile Whirl-Pak bag (Nasco, Modesto, CA, USA), rubbed by hand, and macerated for 2 min. The bacterial population in the buffer was determined by serial dilution in BPW, plating on TSAN and Sorbitol MacConkey sorbitol agar (SMACN, Difco) and incubated at 37 °C for 24 h [39]. In addition to enumeration, BPW samples were enriched according to FDA Bacteriological Analytical Manual enrichment protocol [40]. Briefly, samples were enriched by adding 20 mL of double-strength modified BPW with pyruvate and incubated at 37 °C for 24 h. When counts for the respective samples were negative by direct plating, enrichments were streaked on SMACN and incubated at 37 °C for 24 h. Presumptive colonies isolated from SMACN plates were confirmed as *E. coli* O157 by agglutination assays (*E. coli* O157 latex agglutination test, Microgen Bioproducts Ltd., Surrey, UK)

### 2.9. Real-Time qPCR Assay

To observe differential regulation of bacterial genes in response to attachment on nuts, RT-qPCR was performed. For the real-time assay, hazelnuts were inoculated with *E. coli* O157:H7 Odwalla strain by dipping in sterile bags containing 10 mL of inoculum for a duration of 1 h. The inoculated nuts were then washed in 20 mL of BPW by gently shaking the bag for 30 s. Each nut was washed three times. Bacterial RNA extraction from inoculated hazelnuts was performed according to Barak et al. [41]. Briefly, following washing, hazelnuts were transferred to a sterile 50 mL centrifuge tube containing sterile BPW and sonicated for 1 min at 250 W in a water bath sonicator. The detached cells were pelleted following centrifugation at 45,440× *g* for 45 min at 4 °C. The supernatant was discarded and the pellet was resuspended in 1.5 mL of RNAprotect bacterial reagent (Qiagen, Valencia, CA, USA). Total RNA was extracted using the RNeasy kit (Qiagen, Valencia, CA, USA). Additionally, RNA was extracted from the inoculum used to inoculate the nuts to serve as the comparative baseline in the analysis of differential gene expression. Following RNA extraction, DNase treatment (Promega, Madison, WI, USA) was performed and the samples were stored at −80 °C until use [41]. One microgram of DNA-free RNA was subject to complementary DNA synthesis. cDNA was synthesized using the iscript cDNA synthesis kit (Biorad, Hercules, CA, USA) and used as the template for RT-qPCR. The amplification product was detected using SYBR Green reagents (Biorad, Hercules, CA, USA). Relative gene expression was determined by the comparative critical threshold (2^−∆∆Ct^) value method using a StepOnePlus^TM^ Real-Time PCR system (Applied Biosystems, Carlsbad, CA, USA), and expressed as fold change in expression relative to controls. Primers for genes essential for attachment (*csgA*, *fliA* [42], *escN* [43]) and stress response (*rpoS*; [42]) were evaluated for their differential gene regulation with reference to *gapdh* expression (housekeeping control; [42]).

### 2.10. Statistical Analysis and Modeling Microbial Decline

Six replicates were sampled at each time to enumerate the surviving *E. coli* O157:H7 population and each experiment was repeated two times. When the enumerated bacterial number was below the Limit of Detection (LOD; 10 CFU/nut), but positive following enrichment, an assigned value just below the LOD (9 CFU/nut or 0.9 log CFU/nut) was used for the analysis. Similarly, when samples were negative by enrichment, an assigned value of 0.1 CFU/nut (−0.9 log CFU/nut) was employed [39]. Pooled samples were averaged and the data were analyzed using the mixed procedure of SAS (Statistical Analysis Software, SAS Institute Inc., Cary, NC, USA) ver. 9.2. Differences among the means were detected at *p* < 0.05 using the Fisher’s least significance difference. Best-fit models (Baranyi and Gompertz) were generated using DMFit and were selected based on *R*^2^ values [35].

## 3. Results and Discussion

Tree nuts, in general, have low water activity and, therefore, do not favor bacterial growth. However, different foodborne pathogens, including *Salmonella*, *E. coli* O157, and *Listeria* have been detected on nuts [44,45,46]. Further, *E. coli* O157 was implicated in the 2011 multistate foodborne outbreak that was traced back to contaminated in-shell hazelnuts [8]. Given the potential for the transmission of this pathogen via tree nuts, the present study investigated the ability of *E. coli* O157:H7 to attach and survive on in-shell hazelnuts when stored at ambient conditions.

### 3.1. Survival of E. coli O157:H7 on Hazelnuts

As with fresh produce, harvesting and processing methods can play a significant role in the potential contamination of nuts. For instance, the outbreak strains were isolated from the orchard soil samples in the 2001 and 2002 almond outbreaks [47]. Further, the rate of pathogen isolation was found to increase during the months when harvesting occurred and following a rain event [37,48,49]. Therefore, contamination of nuts can occur via aqueous carriers, such as following a rain event, or through dry carriers, including dust generated during harvesting and husking of hazelnuts [16,18,50]. Hence, in this study, an aqueous and a dry mode of inoculation were followed to evaluate *E. coli* O157 attachment and survival on hazelnuts

In-shell hazelnuts were inoculated with a four-strain *E. coli* O157:H7 cocktail and survival was determined over 12 months (360 days) of storage at 24 ± 1 °C; 40% ± 3% RH (ambient conditions). Following the initial dip inoculation, approximately 7.73 log CFUof *E. coli* O157 was recovered per nut. After inoculation, nuts were placed on filter papers and dried for 24 h. At the end of the drying period, approximately 6.64 log CFU of *E. coli* O157 was recovered per nut. Previous studies have demonstrated a similar reduction in *E. coli* O157 populations on in-shell walnuts and almonds following dip inoculation and drying for 1–3 days [35,39]. With the dry inoculation, sand was inoculated with 10 log CFU/g and dried at 40 °C for 24 h. At the end of drying, approximately 7.8 log CFU/g of *E. coli* O157 was recovered from the sand. Similarly, pathogen recovery levels have been previously reported for *Salmonella* when using sand for dry inoculation of walnut kernels [15]. Inoculated sand was then used to artificially contaminate the in-shell hazelnuts.

**Figure 1:**
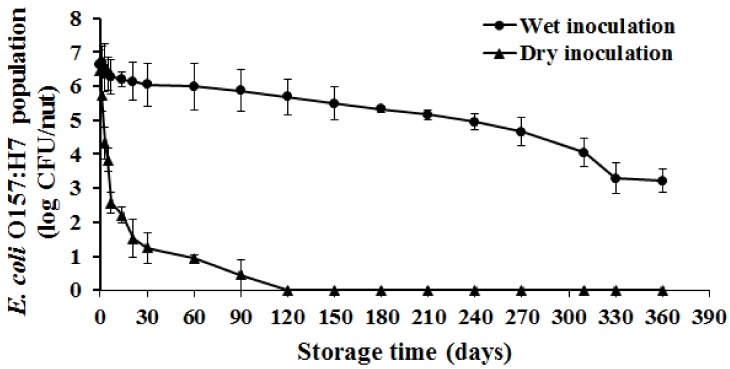
Survival of *E. coli* O157:H7 on in-shell hazelnuts. In-shell hazel nuts were inoculated with approximately 7.7 log CFU of the pathogen/nut by dipping or using a sand carrier. Following inoculation, the nuts were dried for 24 h and stored under ambient conditions for one year. At designated times during the storage, nuts were sampled to enumerate the surviving *E. coli* O157 populations. Data are represented as the mean ± standarad deviation of mean.

At the initial sampling time (immediately prior to storage), approximately 6.4 log CFU of *E. coli* O157:H7 was recovered from the wet and dry inoculated nuts (Figure 1). Irrespective of the inoculation method, a significant reduction in the pathogen population was observed over time (*p* < 0.05). Overall, a 3.4 and 6 log reduction in the *E. coli* O157 population was observed at the end of the study with the wet and dry inoculated samples, respectively. Specifically, a 0.5, 1, and 3 log reduction in pathogen population was observed on day 21, 120, and 330 of storage when using an aqueous carrier. At the end of the 12 month period, a significant population of *E. coli* O157 (3.2 log CFU/nut) was recovered from the wet inoculated nuts (*p* < 0.05). Previous studies investigating the survival of *E. coli* O157 on walnuts, peanut kernels, pecan halves, almond kernels and in-shell pistachios have demonstrated a more rapid decline in *E. coli* O157 populations when stored under ambient temperature [35,38,39]. The recovery of higher pathogen populations in the present study could be due to greater initial population observed in this study (6.4 log CFU/nut) when compared to previous work (5–3.5 log CFU/nut; [35,38,39]). This is in corroboration with an earlier study by Uesugi et al. [37] which demonstrated that inoculum concentration has a significant effect on pathogen survival on nuts over time.

Storage of in-shell nuts inoculated with a dry carrier was also associated with a significant reduction in pathogen population over time (*p* < 0.05). Approximately a 2, 4, and 6 log reduction in pathogen population was observed on the dry inoculated nuts on day 3, 14, and 90 of storage (Figure 1). Further, a rapid decline in pathogen numbers was observed with the dry inoculated nuts when compared to the wet inoculation method. For example, bacterial numbers were below the limit of detection on day 60 of incubation on dry inoculated nuts while approximately 5.9 log CFU/nut was still recovered from the wet inoculated nuts. These results were further validated after being fit to a best-fit model which determined that the pathogen levels declined at a rate of 0.53 log CFU/g/day (*R*^2^ = 0.94) for dry inoculated nuts as opposed to a death rate of 0.01 log CFU/g/day (*R*^2^ = 0.92) when using the wet inoculation method. Additionally, *E. coli* O157 was not recovered in nut samples following enrichment on days 120–360 of the study. These results suggest that *E. coli* O157 survival was significantly impaired when using a dry carrier. However, Blessington et al. [15] did not observe a significant difference in *Salmonella* survival on walnut kernels following wet and dry inoculation.

### 3.2. Attachment of E. coli O157 on In-Shell Hazelnuts

**Figure 2:**
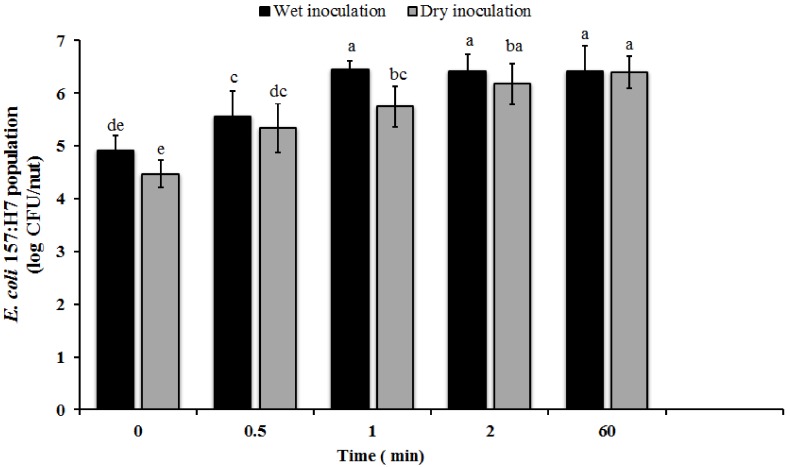
Attachment of *E. coli* O157:H7 on in-shell hazelnuts. In-shell hazel nuts were inoculated with approximately 7.7 log CFU of the pathogen/nut by dipping or using a sand carrier. Following inoculation, the nuts were sampled at 0, 0.5, 1, 2, and 60 min. The nuts were then washed to remove unattached bacteria and the adherent *E. coli* O157 population was enumerated. Data are represented as the mean ± SD. Bars with different superscripts are significantly different at *p* < 0.05.

Since the initial attachment of the pathogen to the epiphytic surface is critical to its ability to survive and persist on the epiphytic surface, this study investigated the ability of *E. coli* O157 to adhere to hazelnuts. Irrespective of the inoculation method, significant numbers of attached *E. coli* O157 was recovered from the nuts at the initial sampling time. Approximately 4.47 and 4.9 log CFU of *E. coli* O157 was recovered from dry and wet inoculated nuts, respectively, almost immediately on contact (Figure 2). Further, a significant increase in the number of adhered bacteria was observed at 30 s and 1 min (*p* < 0.05). An additional increase in inoculum contact time was not associated with a significant increase in pathogen population (*p* > 0.05). Along the same lines, previous studies have demonstrated that significant populations of *E. coli* O157 can attach onto plums and peaches after as little as 30 and 60 s of contact, respectively [26]. Similarly, significant numbers of adhered *E. coli* O157 was recovered from lettuce leaves, arugula leaves, and cut green peppers following 0.25, 1, and 2 h of initial contact [27,30,51]. Altogether, these data demonstrate that, irrespective of the mode of contamination, *E. coli* O157 can rapidly attach to the epiphytic surface, thereby preventing dislodgement and providing a survival advantage to the pathogen.

**Figure 3:**
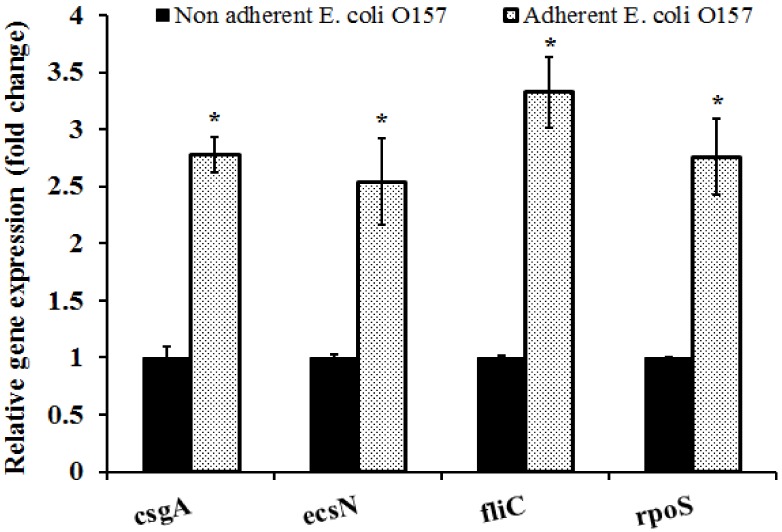
Differences in the expression of adhesin and stress response genes as determined by RTqPCR. Gene expression was assayed using the StepOne Plus Real-Time PCR System. The data were normalized to the endogenous control (Gapdh) and the level of candidate gene expression between *E. coli* O157 cells in the inoculum and those retrieved from the nut surface was compared to study relative gene expression. * Expression of candidate genes in the adherent cells were significantly different from the non-adherent cells at *p* < 0.05.

The attachment of *E. coli* O157:H7 to host, plant, and environmental surfaces is a complex process involving several genetic determinants [52]. For example, mutational studies performed by Matthysse and others [53] demonstrated that cellulose synthesis, colanic acid, and poly-acetylglucosamine (PGA) production-deficient mutants were significantly downregulated in their ability to attach to alfalfa sprouts. Further, using comparative real-time PCR, Carey and others [42] demonstrated that *E. coli* O157 colonization of romaine lettuce resulted in a differential expression of flagellar gene (*fliC*) and common stress regulator (*rpoS*). In addition to *fliC* and *rpoS*, Jeter and Matthysse [54] also identified that deletion of the *csgA* gene that encodes for curli fibres resulted in a significant reduction in the colonization ability of *E. coli* O157:H7 on alfalfa sprouts. Further, deletion of the type III secretion system ATPase (*escN*) was found to significantly inhibit *E. coli* O157 attachment and colonization on arugula and spinach [51,55]. Since RNA extracted from dry inoculated nuts was found to be of low quality and quantity, only wet inoculated nuts were used for the differential gene expression assay in this study. Results of our RT-qPCR assay demonstrate that attachment of *E. coli* O157 to the nuts significantly upregulated the expression of all the genes tested when compared to the control (*p* < 0.05). Genes critical to *E. coli* O157 adhesion and stress response including *csgA*, *escN*, *fliC*, and *rpoS* were upregulated by 2.5- to 3.3-fold following adhesion to the hazelnut shell surface (Figure 3).

## 4. Conclusions

In conclusion, results of our study demonstrate that *E. coli* O157:H7 is adept at attaching and surviving on in-shell hazelnuts when stored under ambient conditions. However, the mode of inoculation or contamination may play a substantial role in determining the epiphytic fitness of the pathogen. Specifically when contaminated by a dry carrier, *E. coli* survival on the shell surface was significantly inhibited when compared to wet inoculation. Overall, these findings highlight the epiphytic fitness of *E. coli* O157 and potential for its transmission by non-traditional, low moisture foods, including nuts.
